# Real-world validation of a reduced-dose continuous infusion cefepime regimen: pharmacodynamic target attainment, efficacy and neurotoxicity

**DOI:** 10.1093/jac/dkag138

**Published:** 2026-04-17

**Authors:** Kieffer Korvin, Sylvain Goutelle, Paul Laffont-Lozes, Fanny Villa, Aurélie Martin, Myriam Chiaruzzi, Paul Loubet, Albert Sotto, Romaric Larcher

**Affiliations:** Department of Infectious and Tropical Diseases, Nimes University Hospital, Nimes, France; CHU de Lyon, Service de Pharmacie, Groupement Hospitalier Nord, Hôpital Pierre Garraud, 136 rue du Commandant Charcot, Lyon cedex 05 69322, France; Laboratoire de Biométrie et Biologie Évolutive, Université Claude Bernard Lyon 1, UMR CNRS 5558, University of Lyon, Bât. Grégor Mendel, 43 bd du 11 Novembre 1918, Villeurbanne Cedex 69622, France; ISPB—Faculté de Pharmacie de Lyon, Université Claude Bernard Lyon 1, University of Lyon, 8 Avenue Rockefeller, Lyon 69003, France; Department of Infectious and Tropical Diseases, Nimes University Hospital, Nimes, France; Department of Pharmacy, Nimes University Hospital, Nimes, France; Department of Infectious and Tropical Diseases, Nimes University Hospital, Nimes, France; Department of Infectious and Tropical Diseases, Nimes University Hospital, Nimes, France; Department of Infectious and Tropical Diseases, Nimes University Hospital, Nimes, France; Department of Infectious and Tropical Diseases, Nimes University Hospital, Nimes, France; VBIC, Univ Montpellier, INSERM, Nimes, France; Department of Infectious and Tropical Diseases, Nimes University Hospital, Nimes, France; VBIC, Univ Montpellier, INSERM, Nimes, France; Department of Infectious and Tropical Diseases, Nimes University Hospital, Nimes, France; PhyMedExp, INSERM, CNRS, Univ Montpellier, Montpellier, France

## Abstract

**Background:**

Cefepime is a key carbapenem-sparing agent due to its stability against AmpC β-lactamases. However, high plasma concentrations are associated with cefepime-induced neurotoxicity (CIN). Following 2019 EUCAST reclassification of ‘intermediate’ as ‘susceptible, increased exposure’, higher doses (6 g/day) are often recommended. Pharmacokinetic/pharmacodynamic (PK/PD) simulations suggest that a reduced daily dose of cefepime 4 g/day administered by continuous infusion may achieve adequate target attainment while limiting toxicity, but real-world clinical data are scarce.

**Methods:**

We conducted a prospective, single-centre observational study including adult inpatients treated with cefepime administered as a 2 g loading dose followed by continuous infusion of 4 g/day. Therapeutic drug monitoring was performed to assess steady-state free cefepime concentrations (ƒCss). The primary endpoint was pharmacodynamic target attainment (100% ƒT > MIC) for EUCAST ‘susceptible, increased exposure’ breakpoints.

**Results:**

Among 46 included patients, median ƒCss was 26.2 mg/L (IQR 18.4–33.2). Pharmacodynamic targets were achieved in 96% of patients for Enterobacterales (MIC 4 mg/L) and 93% for *Pseudomonas aeruginosa* (MIC 8 mg/L). Clinical efficacy was observed in 96% of cases. Signs consistent with CIN occurred in three patients (6.5%), mainly in the context of renal function deterioration or pre-existing neurological vulnerability.

**Conclusion:**

A reduced-dose cefepime regimen consisting of 4 g/day administered by continuous infusion achieves high pharmacodynamic target attainment with a favourable efficacy–toxicity balance in real-life clinical practice. This strategy represents a promising alternative to higher-dose regimens and supports individualized dosing guided by renal function and therapeutic drug monitoring.

## Introduction

Cefepime is a fourth-generation cephalosporin with a broad-spectrum activity that includes coverage of Gram-negative bacilli, such as Enterobacterales and *Pseudomonas aeruginosa*.^1,2^ It is also active against Gram-positive bacteria, including methicillin-sensitive *Staphylococcus aureus* and Streptococci.^3^ A key advantage is its activity against group 3 Enterobacterales with chromosomal class C β-lactamases (AmpC), with cefepime remaining effective even when AmpC is overexpressed.^1–3^ Cefepime’s pharmacokinetic is characterized by predominantly renal elimination, accounting for 85% of its clearance, and a half-life of ∼2 h.^4,5^ This profile makes it particularly relevant for treating severe infections where rapid bactericidal activity is mandatory. Its ability to target difficult-to-treat organisms and its stability against many β-lactamases (including overexpressed AmpC), make it a valuable option in antimicrobial stewardship strategies for sparing carbapenem use.^1,2,6^

The main limitation of its use is the risk of cefepime-induced neurotoxicity (CIN), which is higher than with other β-lactams, thereby restricting its use primarily to the hospital setting.^7,8^ CIN can range from mild alterations in mental status to more severe conditions such as encephalopathy, myoclonus and seizures.^7,8^ Identified risk factors include renal impairment, alterations in blood-brain barrier integrity, and elevated plasma drug levels.^7,9^ Thus, therapeutic drug monitoring (TDM) may help reduce the incidence of CIN.^10^ In this context, PK/PD modelling may also guide the development of dosing regimens that minimize neurotoxicity risk.^11^

A recent PK/PD study used Monte Carlo simulations to explore alternative dosing strategies capable of achieving predefined pharmacodynamic targets (T > MIC) for seven β-lactams categorized as ‘susceptible, increased exposure’ under the European Committee on Antimicrobial Susceptibility Testing (EUCAST) revised clinical breakpoint system.^12^ Notably, the study found that for certain antibiotics, including cefepime, continuous infusion at lower total daily doses achieved target attainment rates similar to those obtained with higher intermittent dosing.^12^ A continuous daily dose of 4 grams of cefepime ensured >50% T > MIC in over 90% of simulated cases for MIC values up to 8 mg/L, a threshold equal to or above the EUCAST breakpoints of 4 mg/L for Enterobacterales and 8 mg/L for *P. aeruginosa*.^13^

This dosing strategy, which is recommended in the French guidelines,^14^ could reduce total daily cefepime exposure while maximizing time above the MIC and may therefore lower the risk of CIN.^11^ However, these findings have not yet been validated in real-life clinical settings.

We aimed to evaluate the probability of target attainment (PTA), efficacy and safety of this optimized cefepime dosing regimen in adult patients hospitalized for proven or suspected Gram-negative bacterial infections.

## Patients and Methods

### Study design and settings

We conducted a prospective single-centre observational cohort study at Nimes University hospital from February 1, 2024, to April 30, 2025.

In this 2094-beds French University Hospital, an infectious diseases (ID) specialist can be consulted 24/7 by physicians in charge of patients with confirmed or suspected infections to initiate or adapt antimicrobial therapy. In 2024, 4498 interdepartmental consultations were provided.

According to the new French guideline,^14^ from February 1, 2024, ID physicians recommended prescribing cefepime as a 2-g loading dose followed by a continuous infusion of 4 g over 24 h (administered as two 2-g syringes over 12 h each) in patients requiring this antibiotic for nosocomial infections. Cefepime was used either as monotherapy or in combination, depending on the clinical context. In line with the local protocol of Nimes University Hospital, ID specialists also recommended performing TDM of plasma steady-state concentrations (Css) after 48 h of treatment to minimize the risk of CIN in patients receiving high-dose cefepime.

### Patients

Consecutive patients aged ≥18 years who received a continuous infusion of cefepime at 4 g/day following a 2-g loading dose administered over 30 min were included in the study. Patients treated with cefepime for <48 h were excluded. Patients without TDM data and those who refused the use of their data were also excluded. For individuals who received multiple courses of cefepime, only the first treatment episode was considered.

### Data collection

Data were collected prospectively during routine clinical care and analysed later without influencing clinical management. Patients’ demographic, clinical and biological data were collected from the hospital electronic medical record. Hospital ward, age, sex, weight, height, body mass index (BMI), medical history, comorbidities (estimated by the Charlson Comorbidity Index),^15^ glomerular filtration rate (GFR) calculated with the CKD-EPI formula,^16^ antibiotics co-administered, antibiotic regimens, reason for antimicrobial treatment, results of microbiological samples, cefepime plasma concentration, occurrence of adverse effects, and clinical outcomes, were collected.

### Cefepime therapeutic drug monitoring

Cefepime plasma concentrations were measured using an ultra-high performance liquid chromatography method adapted from Legrand *et al.*^17^ Free plasma concentrations of cefepime were estimated as 80% of the total plasma concentrations, assuming a protein binding rate of 20%.^18^

### Microbiology

The Department of Microbiology conducted bacterial identification using mass spectrometry Vitek^®^ MS (bioMérieux, Marcy-l’Etoile, France). Microbial samples are prepared and ionized using the MALDI method; then, the obtained mass spectra are compared with a reference library for identification. Antimicrobial susceptibility testing (AST) was performed using the Vitek 2^®^ automated system (AST-N372 card for urinalysis and AST-N233 card for other samples, bioMérieux) and/or the disc diffusion method on Mueller–Hinton agar (Bio-Rad, Hercules, CA, USA) according to the EUCAST guidelines.^13^

### Outcomes

The primary outcome was the proportion of patients in which the cefepime steady-state free concentrations (ƒCss) were above the ‘susceptible, increased exposure’ MIC for *Enterobacterales* (4 mg/L) throughout the whole dosing interval (100%ƒT > MIC).

Secondary outcomes included the proportion of patients in which the cefepime ƒCss were above the ‘susceptible, increased exposure’ MIC for *Pseudomonas aeruginosa* (8 mg/L) throughout the whole dosing interval, and the proportion of those with cefepime ƒCss above four-fold the MIC throughout the whole dosing interval (100%ƒT > 4×MIC, i.e. 16 mg/L for *Enterobarerales* and 32 mg/L for *P. aeruginosa*).^19^ Secondary outcomes also included clinical efficacy and toxicity assessment.

Clinical efficacy was defined as the explicit resolution of symptoms related to the infection that justified cefepime use at hospital discharge, and the absence of septic complication or death during the treatment period. However, if an obvious cause of secondary infection, not treatable with cefepime, was identified, treatment with cefepime was not considered the cause of failure.

Toxicity was defined as the occurrence of signs compatible with CIN during the treatment period (myoclonus, convulsion, altered mental status), which resolved upon discontinuation of the therapy.^20^ Altered mental status may involve one or more of the following: low level of consciousness (drowsiness, stupor, coma), altered attention and awareness (confusion, inattention, disorientation), altered cognition (impaired memory, language, or executive function) and abnormal behaviour or affect (agitation, hallucinations, personality change).

Clinical efficacy and toxicity assessments were independently performed by two investigators (K.K. and R.L.) through review of medical records. Both evaluators were blinded to the results of TDM and PTA analyses. Disagreements were resolved by consensus.

### Statistical analysis

Qualitative data were described as number and percentage and quantitative data as median and interquartile range (IQR) or mean ± standard deviation (SD). We carried out a linear regression, with the outcome variable cefepime free concentration and the explanatory variables age, sex, weight and GFR. These covariates were defined *a priori* based on data from the literature.^3^ The candidate covariates were included in a Least Absolute Shrinkage and Selection Operation (LASSO) penalized regression model. The penalty coefficient (lambda) was chosen to provide an estimation error lower than 1 SD of the minimum error obtained by 10-fold cross-validation, while being as parsimonious as possible. No variable had a coefficient different from 0 with this lambda coefficient.

Analyses were performed using Medistica pvalue.io, a Graphic User Interface to the R statistical analysis software (version 4.5.0) for scientific medical publications 2019–2024 Available on: https://www.pvalue.io/fr. The scatter plot was generated using python version 3.13 and matplotlib version 3.10. All tests were two-sided, and *P* < 0.05 was considered statistically significant.

### Ethics

This study was conducted in accordance with the principles of the Declaration of Helsinki. The study was approved by the Institutional Review Board (IRB) of Nimes University Hospital (IRB No. 25.10.04). In accordance with French regulations governing observational studies (MR004 methodology), written informed consent was waived. Patients were informed of their participation and had the opportunity to refuse the use of their data.

## Results

### Patients

**Figure 1.**
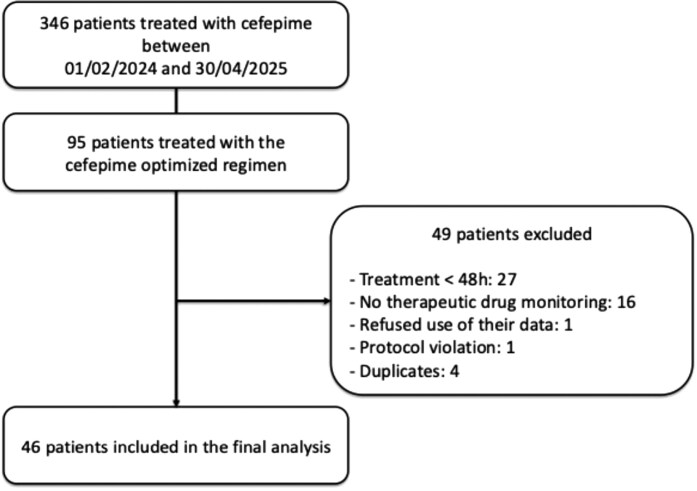
Flow chart.

During the study period, 346 patients were treated with cefepime in our hospital. The cefepime optimized administration regimen was prescribed in 95 patients (27%). Among these patients, 27 received treatment for <48 h, 16 were excluded from the analysis due to the absence of TDM, one patient refuses the use of their data, and one patient who received intermittent infusions was also excluded. Four additional patients were excluded because they had previously received a course of cefepime and had already been included in the study (duplicates). The remaining 46 patients were included in the analysis (Figure 1).

Most of the patients included in the study were men (78%) and the median age was 66-year-old (IQR 59–75). The median GFR and BMI were 84 mL/min/m^2^ (IQR 65–104) and 22 kg/m^2^ (IQR 20–27), respectively. The most common treated diseases were pneumonias (33%), catheter-related bloodstream infections (22%) and intra-abdominal infections (17%). Microbiological samples were mainly positive for group 3 Enterobacterales (*Citrobacter spp., Morganella spp., Enterobacter spp.*) and non-fermenting Gram-negative bacilli (*P. aeruginosa, Acinetobacter spp.*). Patients received cefepime for a median duration of 7 days (IQR 5–8), with TDM performed on day 2 (IQR 2–3) after treatment initiation (Table 1).

**Table 1.** Baseline characteristics

Characteristics (*N* = 46)	*N* (%) or Median (IQR)
Patients	
Age (years)	66 (59–75)
Male sex	36 (78%)
Weight (kg)	66 (60–82)
Height (cm)	174 (168–180)
BMI (kg/m^2^)	22 (20–27)
Charlson comorbidity index	5 (4–6)
GFR (mL/min)	84 (65–104)
≥ 90	18 (39%)
60–90	21 (46%)
30–60	7 (15%)
<30	0 (0%)
Management	
Cefepime duration (days)	6.5 (5–8)
TDM interval (days)	2 (2–3)
Antibiotic combination	15 (33%)
Hospital ward	
Medical	33 (72%)
Surgery	11 (24%)
Rehabilitation	2 (4%)
Intensive care	1 (2%)
Infections	
Pneumonia	15 (33%)
Catheter-related bacteraemia	10 (22%)
Intra-abdominal	8 (17%)
Bone and joint	6 (13%)
Urinary	4 (9%)
Skin and soft tissue	3 (7%)
Microbiological samples	
Blood culture	15 (32%)
Respiratory sample	8 (17%)
Urine culture	3 (6%)
Bone/joint	6 (13%)
Intra-abdominal	4 (9%)
Other	2 (4%)
None	10 (21%)
Pathogens	
Group 3 Enterobacterales	26 (56%)
Non-fermenting bacilli^a^	12 (26%)
Other gram-negative bacilli	3 (7%)
*Staphylococcus aureus*	1 (2%)
Polymicrobial	20 (43%)
Unknown	10 (22%)

BMI, body mass index; GFR, glomerular filtration rate; IQR, interquartile range; TDM, therapeutic drug monitoring.

^a^ *Pseudomonas aeruginosa* (*N* = 8) and *Acinetobacter* spp. (*N* = 4).

### Therapeutic drug monitoring of cefepime

Among the 46 included patients, the median cefepime Css was 32.8 mg/L (IQR 22.9–41.6) and the mean Css was 33.4 mg/L ± 16.1 mg/L, which corresponds to median and mean cefepime ƒCss of 26.2 mg/L (IQR 18.4–33.2) and 26.7 ± 12.9 mg/L.

**Figure 2.**
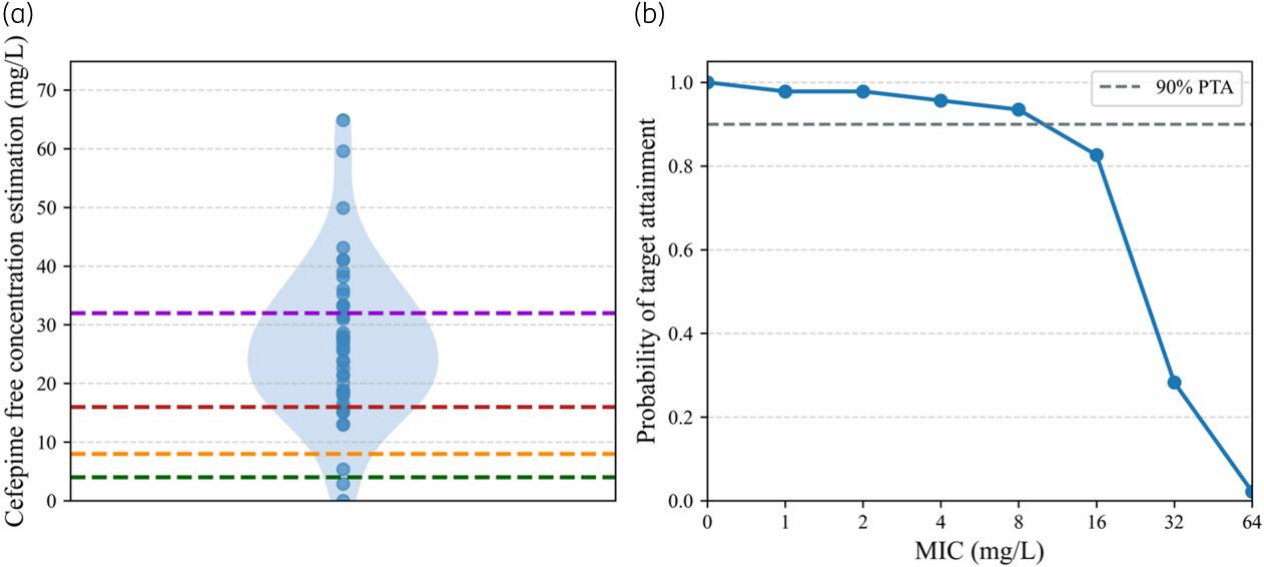
Cefepime exposure and PTA. Distribution of estimated free cefepime plasma concentrations represented as a violin plot, with individual observations overlaid (*N* = 46, blue dots). Dashed horizontal lines indicate pharmacodynamic targets based on worst minimum inhibitory concentration (MIC) values (breakpoint): 4 mg/L for *Enterobacterales* (green), 8 mg/L for *Pseudomonas aeruginosa* (orange), 16 mg/L corresponding to 4×MIC for *Enterobacterales* (red), and 32 mg/L corresponding to 4×MIC for *P. aeruginosa* (purple). (B) PTA according to MIC values. The dashed horizontal line represents the 90% PTA target.

Importantly, 95.7%, 95% CI [85.5%; 99.5%] of the patients (*N* = 44) reached the therapeutic target of 100%ƒT > MIC for *Enterobacterales* and 93.5%, 95% CI [82.1%; 98.6%] (*N* = 43) for *P. aeruginosa*. Moreover, 82.6%, 95% CI [69.3%; 90.9%] of patients (*N* = 38) had reached 100%ƒT > 4×MIC for *Enterobacterales* and 28.3%, 95% CI [15.6%; 40.3%] (*N* = 13) for *P. aeruginosa* (Figure 2 and Table 2).

**Table 2.** Cefepime plasma steady-state concentration (Css) measurements and free concentration (ƒCss) estimation among the study population (*N* = 46)

	Cefepime Css (mg/L)	Cefepime ƒCss (mg/L)
Mean ± SD	33.4 ± 16.1	26.7 ± 12.9
Median (IQR)	32.8 (22.9–41.6)	26.2 (18.4–33.2)

Css, steady-state concentration; ƒCss, free steady-state concentration; IQR, interquartile range; SD, standard deviation.

### Clinical outcomes

Patients treated with the optimized regimen showed a high rate of clinical efficacy (95.7%, 95% CI [85.5%; 99.5%]). Among the two patients who experienced treatment failure, one died from decompensation of a severe underlying cardiomyopathy, progressing to refractory cardiogenic shock 19 days after initiation of antimicrobial therapy while still receiving cefepime (30-day mortality rate in the whole cohort: 2.2%). The other, who was severely immunocompromised, had an undocumented pneumonia and remained febrile on day 3 of treatment, requiring escalation of antibiotic therapy.

Signs of CIN were observed in three patients, 6.5% IC95% [2.2 %; 17.6 %] of the cohort (Table 3). An acute renal failure occurred during therapy in two of them, and unfortunately the cefepime dosage regimen was not adjusted on the decreased renal clearance. The third patient had a history of mild neurocognitive disorder, primarily characterized by memory impairment. Among patients with CIN the cefepime ƒCss were 43.1 mg/L, 38.2 mg/L and 20.1 mg/L, respectively. No patient developed clinically apparent seizures or severe neurological impairments during the treatment period.

**Table 3.** Treatment outcomes and CIN

Patients treated with cefepime (*N* = 46)	*N* (%)
Clinical efficacy	44 (96%)
Treatment failure	2 (4%)
*Persistence/recurrence of infection*	1 (2%)
*Death occurring under antibiotic treatment*	1 (2%)
CIN	3 (6%)
*Altered mental status*	2 (4%)
*Myoclonus*	1 (2%)

### Factors influencing cefepime steady-state free concentration (ƒCss)

In univariable analysis, sex (*P* < 0.01) and GFR (*P* < 0.01) were associated with changes in cefepime ƒCss, whereas age (*P* = 0.59) and body weight were not.

**Figure 3.**
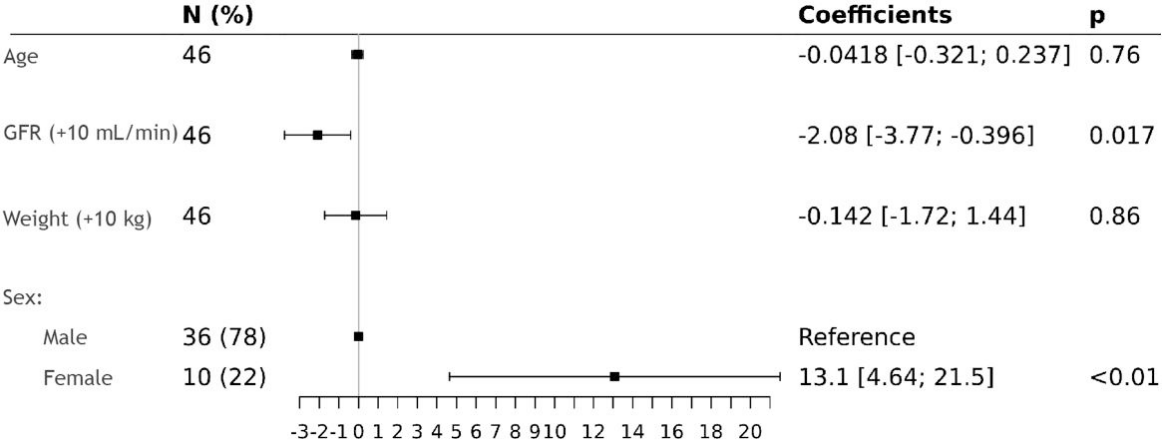
Forest plot showing the results of the multivariable linear regression analysis assessing factors associated with free cefepime concentrations (ƒCss). Squares represent estimated regression coefficients and horizontal bars indicate 95% confidence intervals. Continuous variables are expressed per unit increase (age in years, GFR per +10 mL/min, weight per +10 kg). Sex is included as a categorical variable, with males as the reference category. *P* values indicate the statistical significance of each covariate.

In multivariable analysis we found that cefepime ƒCss remained significantly related to GFR and sex. When GFR increased by 10 mL/min cefepime ƒCss decreases on average by −2.08 mg/L; *P* = 0.017. In addition, we showed that cefepime ƒCss in female patients was on average superior of 13.1 mg/L to cefepime ƒCss in male, *P* ≤ 0.01 (Figure 3).

## Discussion

In this prospective cohort study including 46 patients hospitalized and treated for sepsis with a 2 g loading dose followed by a continuous infusion of 4 g per day of cefepime, we found that plasma cefepime ƒCss were above the EUCAST's ‘susceptible, increased exposure’ MIC breakpoints for Enterobacterales and *P. aeruginosa* in more than 90% of cases. Our findings demonstrate that the optimized dosage regimen, derived from Monte Carlo simulations, achieved the therapeutic target of 100% ƒT > MIC in nearly all patients while limiting the incidence of CIN under 10%. The studied dosing regimen appeared to be effective and safe in our cohort, although these findings should be interpreted cautiously given the observational design, the relatively small sample size, and the limited systematic monitoring for adverse events.

Our results align with those reported by Vercheval and colleagues,^21^ who observed a mean Css of 46.4 mg/L (estimated ƒCss at 37.3 mg/L) in a large retrospective cohort of 98 patients treated with a continuous infusion of cefepime at 4 grams per day. Interestingly, neurotoxicity was more frequent in their cohort, occurring in 16.7% of cases; however, patients in their study had a lower GFR compared with ours (50 versus 80 mL/min). Additionally, recent data indicate that 80% of patients receiving a continuous infusion regimen of cefepime at 6 grams per day had supratherapeutic concentrations, with a mean Css of 59.8 mg/L.^22^ Thus, these findings support that the continuous infusion regimen of 4 grams per day, extrapolated from Monte Carlo simulations, is sufficient to achieve therapeutic targets in most clinical scenarios. Furthermore, this regimen may also reduce the incidence of neurotoxicity when utilized in patients with preserved or mildly impaired renal function.

There is significant concern regarding CIN, and our results are encouraging, demonstrating a low incidence at 6.5%. Indeed, with CIN reported rates up to 23%,^7,20,23,24^ cefepime is sometimes underutilized due to apprehension about neurotoxicity; thus, a safe and effective dosage regimen may encourage broader clinical use. Two out of the three patients in our cohort who exhibited signs of CIN had acute renal impairment without dosage adjustment—a scenario frequently identified in the literature as a major risk factor.^25^ The third patient presented pre-existing neurocognitive impairment, another well-recognized predisposing factor.^7^

Although it is widely recognized that higher cefepime exposure increases the risk of CIN, the precise relationship between cefepime concentrations and its occurrence remains incompletely understood. To date, mainly through concentrations have been used for the evaluation of CIN and the toxicity thresholds reported in the literature vary between 7.7 and 40 mg/L,^23,26,27^ with sensitivities and specificities for CIN prediction differing considerably among studies.^11^ Actually, the underlying pathophysiological mechanism of CIN has not been fully elucidated, but it appears to involve a concentration-dependent blockade of γ-aminobutyric acid (GABA)_A_-receptors.^25,28^ Thus, CIN may actually be more closely related to peak concentration and overall daily exposure rather than through concentration. It can therefore be expected that reducing the daily dose and avoiding peak concentrations by using continuous infusion may help limit toxicity. However, the relationship between Css and CIN is not clearly established in the literature, as the proposed toxicity thresholds vary from 33.8 to 63.8 mg/L,^21–23^ making their use difficult in clinical practice.^8,29,30^ Moreover, as illustrated by our findings, patients who develop CIN tend to have higher plasma concentrations compared with the rest of the cohort; but some patients exhibit markedly higher levels without any signs of CIN, highlighting the interindividual variability in CIN susceptibility.^7,8,9^

One of the strengths of our study is that it demonstrates that, despite a low incidence of CIN—likely related to a reduction in total daily dose—we achieved high cefepime ƒCss, which were associated with a favourable outcome in 96% of patients in the cohort. Our results encourage the use of continuous infusion of cefepime over intermittent infusion as it has been reported for beta-lactam antibiotics in recent studies.^31^ Continuous infusion, when combined with an appropriate loading dose, allows rapid attainment and sustained maintenance of ƒCss above the MIC of suspected or documented pathogens, thereby achieving 100% ƒT > MIC.^32^ Our study confirms that by systematically administering a loading dose, patients rapidly achieve pharmacokinetic targets, as demonstrated by our early cefepime TDM. In contrast, in the study by Vercheval *et al*.,^21^ only two-thirds of patients received a loading dose and TDM was performed later, resulting in more heterogeneity and limiting the generalizability of the findings from that cohort. However, our results also confirmed the need for caution in patients with altered renal function and in women.^25,33^

This study has several limitations. First, the relatively small sample size and the single-centre design may limit the generalizability of the findings, particularly in specific subgroups such as patients with renal impairment. In addition, the observational design precludes establishing a causal relationship between drug exposure and clinical outcomes, including efficacy and neurotoxicity. Second, the assessment of CIN was performed by the treating clinicians or the consulting ID physicians, without the use of a standardized scoring system, which may have introduced classification bias due to interobserver variability. In addition, EEG monitoring was not systematically performed; therefore, subclinical neurotoxicity, such as non-convulsive seizures or encephalopathy, cannot be excluded, and the true incidence of CIN may have been underestimated. Third, although continuous infusion provides several advantages, it also presents significant risks if not properly managed. Failure to achieve the target Css can result in 0% ƒT > MIC, leading to subtherapeutic exposure and high-risk of treatment failure. This highlights the essential role of TDM to ensure efficacy and minimize the risk of underexposure. Fourth, late TDM was not performed in our study, which limits the ability to assess potential drug accumulation over time, particularly in patients with fluctuating renal function. Nonetheless, patients were clinically monitored throughout the treatment course, and no cases of delayed-onset CIN were observed. Finally, our cohort includes only one ICU patient, and the fact that only 28% achieved 100% ƒT > 4×MIC for the treatment of *P. aeruginosa* should warrant caution when considering the use of this dosing regimen in critical care settings. Indeed, several authors suggested targeting 100%ƒT > 4-6×MIC, to improve microbiological eradication,^34^ and clinical outcomes,^34–37^ and reduce emergence of antimicrobial resistance^37,38^ in the critically ills. However, achieving these targets is sometimes limited by toxicity-related issues.^21–23^

In conclusion, we demonstrate, in a prospective real-life study, that a cefepime regimen consisting of a 2 g loading dose followed by a continuous infusion of 4 g per day achieved 100% ƒT > MIC for Enterobacterales and *P. aeruginosa* in nearly all patients, with 96% attaining clinical efficacy and a low incidence of CIN. These results provide real-world validation of the French national recommendations derived from simulation-based data.

However, considering the narrow therapeutic index of cefepime and the substantial interindividual variability in drug pharmacokinetics, individualized dosing—particularly guided by renal function and sex—alongside systematic TDM, is essential to optimize efficacy while minimizing the risk of toxicity. Our findings also underscore the limitations of applying this dosing strategy in critically ill patients, especially in clinical scenario where higher exposure targets may be required. Further multicentre studies are warranted to confirm these results and to refine cefepime dosing strategies in high-risk clinical settings.

## Data Availability

The authors consent to share the collected data with others. Data will made available by the authors, without undue reservation, immediately after the main publication and indefinitely.
